# A rare case of jejunal atresia

**DOI:** 10.1016/j.ijscr.2021.106714

**Published:** 2021-12-28

**Authors:** Jayalaxmi Shripati Aihole

**Affiliations:** Dept of Paediatric surgery, Indira Gandhi Institute of Child Health, Bengaluru, Karnataka, India

**Keywords:** Jejunal atresia, Anastomosis, Excisional tapering

## Abstract

**Introduction:**

Intestinal atresia more common in the small bowel, apart from large intestine. Jejunal atresia characterized by complete occlusion of the intestinal lumen, is a rare congenital anomaly occurring in 1 in 12,000 live births.

**Importance:**

The jejunal atresia can be single or multiple occurring anywhere from the ligament of Treitz to the jejuno-ileal junction, requiring immediate surgical attention to prevent mortality and morbidity among these neonates.

**Case presentation:**

A rare case of jejunal atresia in neonate and its management has been discussed here.

**Clinical discussion:**

Surgical excision of the involved bowel and end to end anastomosis of the normal bowel is definitive treatment.

**Conclusion:**

The morbidity associated with post-operative hypo persistaltic bowel can be minimised by adding oral prokinetics in controlled manner.

## Highlights

• The jejunal atresia can be single or multiple occurring anywhere from the ligament of Treitz to the jejuno-ileal junction.
• Because of the range of the mobility of jejunum and ileum, as well as due to their anatomy of the vascular arcade their potential for compression and volvulus is high, hence vascular ischemia.
• Type IV jejunal atresia comprising of multiple atretic segments of jejunum. Only few cases have been reported so far
• Patients with jejunoileal atresia usually have prolonged ileus after surgical correction for upto 3to 4 weeks.depending on total parenteral nutrition for long time.
• to reduce this intestinal hypomotility, prokinetics have been suggested like neostigmine and erythromycin apart from excisional tapering of the proximal hypoperistaltic jejunum.

## 1 Introduction

Suggested possible aetiologies of intestinal atresia includes, an antenatal ischemic event causing necrosis and resorption of the involved bowel. Because of the range of the mobility of jejunum and ileum, as well as due to their anatomy of the vascular arcade their potential for compression and volvulus is high, hence resulting in vascular ischemia.

## 2 Case summary

Day one female baby born to a gravid 3, para 3, mother by full term vaginal delivery without any significant antenatal history to a nonconsanguineously married couple.

**Fig. 1:**
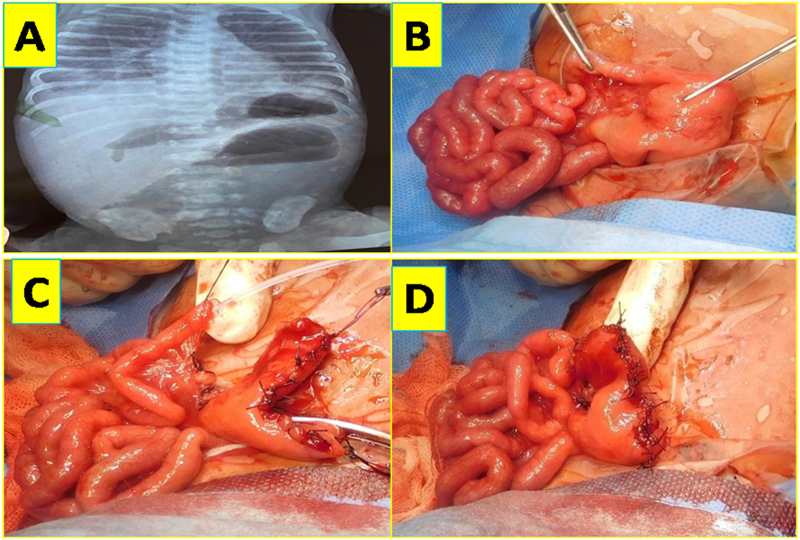
A-Erect abdomen radiography showing step ladder bowel gas pattern. B-Intra operative appearance of type 4 jejunal atresia. C-Excisonal tapering of the proximal jejunum after resection of atretic segment. D-End to end jejunojejunal anatsomosis.

**Fig. 2:**
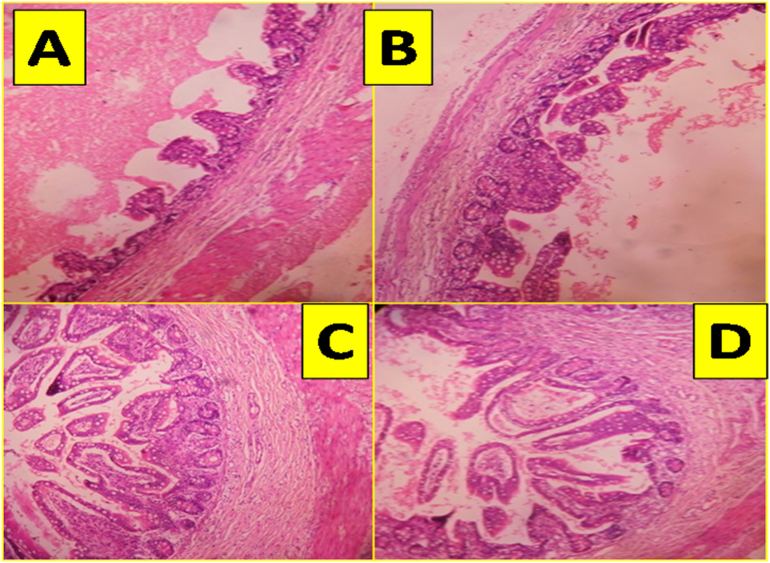
Histopatholgical pictures-low power field (10×). A-and B—showing atretic jejunal segment with atrophic intestinal wall. C and D-Normal jejunal wall layers.

Baby was referred to us in view of abdominal distension, bilious vomiting and not passed meconium. Erect abdomen radiography showing step ladder pattern of bowel gas shadows without rectal gas shadow (Fig 1A). After initial resuscitation, exploratory laparotomy revealed; type 4 jejunal atresia involving middle of jejunum 12 cm from duodeno jejunal flexure for a length of around 12 cm with string of beads appearance having rest of jejunum, ileum and colon essentially normal and had normal bowel length. Excisional tapering of proximal dilated jejunum of around 6 cm was done and atretic jejunum was excised, end to end anastomosis of jejunum was done (Fig. 1B, C, D). Post operatively baby had hypoperistaltic bowel with abdominal distension, hence 1 mg injection neostigmine was started through nasogastric tube with 5 ml normal saline initially followed by dilution with breast milk later on, three times a day. Baby responded well, started passing cholic stools and gradually direct breast feeds were established and baby was discharged. Baby's cardiac, genetic and other systemic evaluations were essentially normal. Histopathology of the resected atretic segment of jejunum revealed atrophic or aborted intestinal epithelium with thinned or atrophic layers of jejunal wall (Fig. 2E, F, G, H).

## 3 Discussion

As per Louw and Bernard's classic studies, late intrauterine mesenteric vascular failure or accident, is the key factor for most of the small intestine and colonic atresia. However other studies hypothesize that, small bowel atresia occurs as a result of intestinal volvulus, intussusceptions, internal hernia, and tight gastroschisis or omphalocele defect. However, jejunal atresia has been reported to be associated with volvulus without having mesenteric vascular anomaly. Favara et al. also suggested antenatal vascular accident as causative factor for intestinal atresia [1], [2].

There are five standard types of atresia as described Grosfeld and Louw et al. Type I atresia constitute luminal webs or membranes with mural continuity. Type II atresia is characterized by blind ends joined by fibrous cord. Type IIIa atresia has two disconnected ends without a fibrous cord and a mesenteric gap. Type IIIb atresia has two separated ends along with a large mesenteric defect, complete absence of distal small intestine mesentery with proximal jejunal atresia, also called “Christmas tree” or “apple peel” atresia. Type IV atresia is characterized by multiple segments of atresia. and type V is stenosis [1], [2], [3].

Our patient had a Type IV jejunal atresia comprising of multiple atretic segments of jejunum. Only few cases have been reported so far.

Jejuno-ileal atresias can be associated with prematurity (50%), polyhydramnios (25%), cystic fibrosis (20%), gastroschisis and short gut syndrome [1], [2], [3], [4].

Bilious vomiting, increasing abdominal distension, failure to pass meconium are the main presenting symptoms [1], [2], [3], [4].

The classic radiographic sign of jejunal atresia is that of a triple bubble appearance for a proximal obstruction, however there can be multiple dilated small bowel loops proximal to the atresia and the number of dilated loops increase as the point of atresia becomes more distal as was seen in this case.

The goal in treatment of intestinal atresia is to resect the atretic segment and establish intestinal continuity with as much functional bowel as possible [1], [2], [3], [4], [5].

Patients with jejunoileal atresia usually have prolonged ileus after surgical correction for up to 3to 4 weeks.depending upon total parenteral nutrition (PN). This prolonged dependency on PN, can manifests as hepatic steatosis, cholestasis, fibrosis, and even progression to cirrhosis and end-stage liver disease. Hence to reduce this intestinal hypomotility, prokinetics have been suggested like neostigmine and erythromycin [3], [4], [5].

Author had one day old neonate with type 4 jejunal atresia which was managed by resection and excision tapering of the proximal jejunum for about 7 cm and end to end anastomosis. Post operatively baby was started on neostigmine along with erythromycin, due to hypo peristaltic the bowel and continued 2 weeks thereafter, gradually tapering and subsequently stopping the drug without any side effects. Baby is now two years old and doing well.

The work has been reported in line with the SCARE 2020 Criteria [6].

## Provenance and peer review

Not commissioned, externally peer-reviewed

## Sources of funding

None.

## Ethical approval

Yes. Taken from IRB of IGICH.

## Consent

Yes.

## Author contribution

Dr Jayalaxmi Shripati Aihole.

## Research registration studies

None.

## Guarantor

Dr Jayalaxmi Shripati Aihole.

## Declaration of competing interest

None.
